# Mental health difficulties and related factors in Chinese children and adolescents during the COVID-19 pandemic: a cross-sectional study

**DOI:** 10.1016/j.jped.2024.03.004

**Published:** 2024-04-18

**Authors:** Tongtong Li, Chunhao Li, Guoquan Zhang, Naijian Zhang, Jing Li, Qinghan Ren, Wen Li, Zhenshu Li, Guowei Huang, Jing Yan

**Affiliations:** a Tianjin Medical University, School of Public Health, Tianjin 300070, China; b Tianjin Key Laboratory of Environment, Nutrition and Public Health, Tianjin 300070, China; c Southern Medical University, School of Public Health, Guangzhou, Guangdong 510515, China; d Tianjin Medical University Eye Hospital, Eye Institute and School of Optometry, Tianjin Branch of National Clinical Research Center for Ocular Disease, Tianjin Key Laboratory of Retinal Functions and Diseases, Tianjin 300384, China

**Keywords:** Mental health, Children, Adolescents, Screen time, Cross-sectional study, COVID-19

## Abstract

**Objective:**

To examine the mental health status and related factors in children and adolescents, and to assess age groups and sexes differences in factors influencing mental health.

**Methods:**

This cross-sectional study was performed on Chinese children aged 6-18 years from November 2021 to January 2022. Mental health difficulties were accessed by the Strengths and Difficulties Questionnaire. Multivariate logistic regression was used to analyze factors associated with mental health status. Multiple linear regression was used to evaluate factors associated with the scores of the Strengths and Difficulties Questionnaire.

**Results:**

The prevalence of mental health difficulties was 12.98% (n =1348). Age (OR, 0.909, [95%CI, 0.830-0.996]), sex (OR, 1.424, [95%CI, 1.033-1.963]) and screen time on weekdays (“≥2” h/d vs “< 1” h/d: OR, 2.001, [95%CI, 1.300-3.080]) were related factors for mental health difficulties. For children (year ≤ 12), the strongest related factor for mental health difficulties was screen time on weekdays (“≥ 2” h/d vs “< 1” h/d: OR, 1.821 [95%CI, 1.203-2.755]). The risk of mental health difficulties in females with ≥ 2 h/d screen time on weekends was 3.420 times higher than those with < 1 h/d (OR, 3.420, [95%CI, 1.923-6.081]).

**Conclusion:**

The prevalence of mental health difficulties among children and adolescents was relatively high. The lower age, female sex and excessive screen time were associated with a higher risk of mental health difficulties. The factors influencing mental health varied by different age groups and sexes. Thus, specific measures for different age groups and sexes should be adopted to mitigate the impact.

## Introduction

Mental health problems are common and have become a major disease burden among children and adolescents. Approximately 10–20% of children and adolescents worldwide suffer from mental health problems, which have become the leading cause of the subsequent development of psychological disorders including eating disorders, risk-taking behaviors, and self-harm or even suicide.^1^^,^^2^ These problems have the potential to disrupt education and employment. According to the “Report on National Mental Health Development in China (2019-2020)”, the detection rate of depression among primary school students was about 10%, while that in high school students reached 40%. Children and adolescents are the critical stage of individual physical and mental health development, which is the key to ensuring mental health in adulthood.^3^^,^^4^

On January 31, 2020, the World Health Organization declared that the outbreak of coronavirus disease 2019 (COVID-19) constituted a Public Health Emergency of International Concern (PHEIC).^5^ In response to the COVID-19 outbreak, the Chinese government has ordered a nationwide school closure as an emergency measure to prevent the spreading of the infection. Public activities were discouraged. The Ministry of Education estimated that more than 180 million primary and secondary students were confined to their homes.^6^ At the end of January 2020, students completed distance education by online courses at home. It was speculated that the closure of schools and studying at home may have negative effects on children's physical and mental health.^7^ Psychological impact on children and adolescents was an important but easily neglected issue. Stressors such as fears of infection, lack of in-person contact with classmates, friends and teachers, limited outdoor activities and increased screen time can have even more problematic and enduring effects on children and adolescents.^8^

Beyond mental health problems associated with home isolation, evidence suggested that the influence of various factors such as sex, residence, single and non-single child, educational level of parents and socioeconomic status on mental health status.^9^^,^^10^ Identification of modifiable risk factors remains a fundamental and cost-effective strategy in reducing the number of individuals affected by mental health problems. However, most studies to date focused on the relationship between related factors and mental health status among the entire population of children and adolescents.^11^, ^12^, ^13^ Less research has been performed on age groups and sexes difference in mental health problems.

To fill this gap, the aim of the present study was to investigate the mental health status and related factors in Chinese children and adolescents during the COVID-19 pandemic, and to assess age groups and sex differences in mental health status. The present findings may provide evidence for developing tailored mental health problem prevention strategies in China during and beyond the COVID-19 pandemic.

## Methods

### Study population

This cross-sectional study was performed on Chinese children and adolescents aged 6-18 years in primary and secondary school from November 2021 to January 2022. Study participants were from northern China, including Tianjin, Hebei and Shandong Provinces. Inclusion criteria: 1) primary and secondary school students aged 6 to 18 years old; 2) questionnaires completed by students themselves or their parents; 3) questionnaires with complete information. A total of 1684 eligible participants were recruited, but individuals were excluded due to abnormal body mass index (BMI) range (30 participants) and missing data (306 participants). In the end, 1348 participants were included in the final analysis (80.0% participation rate) (Supplementary Fig. 1).

The study complied with the principles of the Declaration of Helsinki and was approved by the Ethics Committee of Tianjin Medical University, China (study number: TMUhMEC2021001). In addition, all participants were aware of the research purpose and provided written informed consent.

### The general health questionnaire

A face-to-face interview was performed to collect information including sociodemographic characteristics and behavior and lifestyle by general health questionnaire. Sociodemographic characteristics included age, sex, weight, height, residence, only child, household income per month and parents’ education level. The two options for residence were urban and rural areas. Levels of parents’ education were categorized as “junior high school and below”, “senior high school” and “university and above”. Household income (i.e., the total monthly income for family members) was categorized into three groups: < 3000 Chinese Yuan (CNY), 3000-10000 CNY and > 10000 CNY. Behavior and lifestyle included BMI, sleep duration, outdoor exercise time per day (< 1 hour, 1-2 hours, ≥ 2 hours), screen time on weekdays (< 1 hour, 1-2 hours, ≥ 2 hours), and screen time on weekends (< 1 hour, 1-2 hours, ≥ 2 hours). BMI was calculated as body weight in kilograms divided by squared standing height in meters (kg/m^2^).

### The Strengths and Difficulties Questionnaire

Mental health difficulties were assessed by the Strengths and Difficulties Questionnaire (SDQ), which has been used to evaluate behavioral and emotional problems in children and adolescents.^14^ Three versions of the SDQ included the SDQ for self-completion, the SDQ for parents, and the SDQ for teachers. The SDQ for self-completion and the SDQ for parents were used in this study. The SDQ for self-completion was filled in by participants aged 11-18 years old according to emotions and behaviors during the past 3 months. The SDQ for parents was filled in according to the behavior and emotion of 4-18 years old participants evaluated by their parents during the past 6 months. The SDQ consisted of 25 items distributed on five scales each: emotional problems, conduct problems, hyperactivity, peer problems, and prosocial behaviors. The items were rated on a 3-point scale (0 = Not true, 1 = Somewhat true, 2 = Certainly true). In addition, the scores from emotional problems, conduct problems, hyperactivity, and peer problems were summed to provide a total difficulties score that ranged from 0 to 40, with higher scores indicating greater difficulties. Conversely, the higher the score of prosocial behaviors, the stronger the social ability.

For the SDQ self-completion, the corresponding scores between 20-40, 7-10, 5-10, 7-10, 6-10, and 0-4 were considered mental health difficulties, emotional problems, conduct problems, hyperactivity, peer problems, and abnormal prosocial behaviors, respectively.^15^^,^^16^ For the SDQ parent version, the corresponding scores between 17-40, 5-10, 4-10, 7-10, 4-10, and 0-4 were considered mental health difficulties, emotional problems, conduct problems, hyperactivity, peer problems, and abnormal prosocial behaviors, respectively.^17^ The Cronbach's alpha of the total scale was 0.678 in this study.

### Statistical analysis

SPSS version 24.0 software (SPSS Inc, Chicago, IL, USA) was used to analyze all data. All continuous variables were described by mean and standard deviation (SD). Categorical variables were presented as numbers and percentages and compared using a chi-squared test between mental health difficulties and mental health. Continuous variables were compared between the groups using independent samples *t*-test if normality assumptions were not met, using Wilcoxon's rank sum test.

To further examine potential factors associated with mental health difficulties, a logistic regression analysis was used, and odd ratios (ORs) with 95% confidence intervals (CIs) were calculated. In addition, multiple linear regression was used to evaluate factors associated with the scores of the SDQ. Test of statistical significance were two-sided and *p <* 0.05 was considered statistically significant.

## Results

### Characteristics according to the risk of mental health difficulties

A total of 1348 participants were included in this study. The age range was 6-18 years old with a mean (SD) age of 11.10 (1.76) years. Of the participants, 726 (53.8%) were boys and 622 (46.2%) were girls. The prevalence of mental health difficulties was 12.98%. Statistically significant differences between the two groups including no mental health difficulties and mental health difficulties were found for different sexes and screen time (*p <* 0.05) (Table 1).

**Table 1:** Characteristics of participants with and without mental health difficulties.

				Mental health difficulties		
Characteristics		Total (n = 1348)		Yes (*n* = 175)	No (*n* = 1173)	*P*
Age, mean (SD), y		11.10 (1.76)		10.93 (1.85)	11.12 (1.75)	0.127
Sleep duration, mean (SD), h/d		9.19 (0.89)		9.25 (0.97)	9.18 (0.87)	0.380
Sex, No. (%)						0.031
Male		726 (53.8)		81 (11.2)	645 (88.8)	
Female		622 (46.2)		94 (15.1)	528 (84.9)	
BMI, No. (%), kg/m^2^						0.124
< 24.0		945 (70.1)		114 (12.1)	831 (87.9)	
≥ 24.0		403 (29.9)		61 (15.1)	342 (84.9)	
Only child, No. (%)						0.292
Yes		328 (24.3)		37 (11.3)	291 (88.1)	
No		1020 (75.7)		138 (13.5)	882 (86.5)	
Residence, No. (%)						0.695
Rural		775 (57.5)		103 (13.3)	672 (86.7)	
Urban		573 (42.5)		72 (12.6)	501 (87.4)	
Paternal education, No. (%)						0.385
Junior high school and below		673 (49.9)		92 (13.7)	581 (86.3)	
Senior high school		414 (30.7)		53 (12.8)	361 (87.2)	
University and above		261 (19.4)		30 (11.5)	231 (88.5)	
Maternal education, No. (%)						0.505
Junior high school and below		721 (53.5)		97 (13.5)	624 (86.5)	
Senior high school		346 (25.7)		45 (13.0)	301 (87.0)	
University and above		281 (20.8)		33 (11.7)	248 (88.3)	
Household income, No. (%), CNY						0.592
< 3000		154 (11.4)		22 (14.3)	132 (85.7)	
3000-10000		927 (68.8)		113 (12.2)	814 (87.8)	
> 10000		267 (19.8)		40 (15.0)	227 (85.0)	
Outdoor exercise time, No. (%), h/d						0.241
< 1		415 (30.8)		65 (15.7)	350 (84.3)	
1-		532 (39.5)		58 (10.9)	474 (89.1)	
≥ 2		401 (29.7)		52 (13.0)	349 (87.0)	
Screen time on weekdays, No. (%), h/d						0.006
< 1		836 (62.0)		94 (11.2)	742 (88.8)	
1-		323 (24.0)		45 (13.9)	278 (86.1)	
≥ 2		189 (14.0)		36 (19.0)	153 (81.0)	
Screen time on weekends, No. (%), h/d						0.021
< 1		547 (40.6)		56 (10.2)	491 (89.8)	
1-		408 (30.3)		60 (14.7)	348 (85.3)	
≥ 2		393 (29.1)		59 (15.0)	334 (85.0)	

BMI, body mass index; CNY, Chinese Yuan.

### Associations between mental health difficulties and effect factors

**Figure 1:**
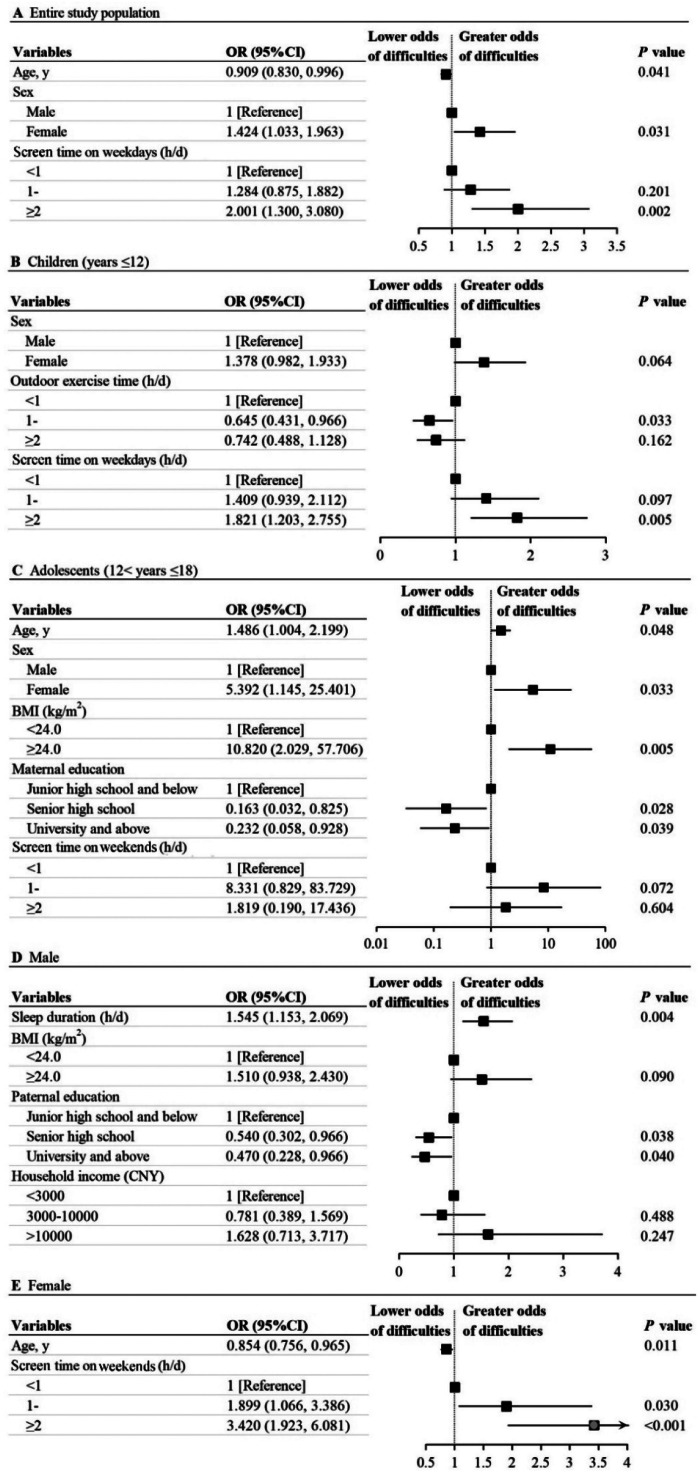
Associations between mental health difficulties and influencing factors in (A) the entire study population, (B) the children, (C) the adolescents, (D) the male, (E) the female.

The result of the logistic regression analysis indicated that age (OR, 0.909, [95%CI, 0.830-0.996]), sex (female vs male: OR, 1.424, [95%CI, 1.033-1.963]) and ≥ 2 h/d screen time on weekdays (“≥ 2” h/d vs “< 1” h/d: OR, 2.001, [95%CI, 1.300-3.080]) were significantly associated with mental health difficulties (Figure 1A).

Interestingly, the factors associated with mental health difficulties were completely different between children and adolescents. For children (years ≤ 12), < 1 h/d outdoor exercise time and ≥2 h/d screen time on weekdays were significantly associated with greater odds of mental health difficulties. The strongest influencing factor for mental health difficulties was screen time on weekdays with two or more hours per day compared to screen time with less than 1 hour (OR, 1.821 [95%CI, 1.203-2.755]) (Figure 1B). For adolescents (12 < years ≤ 18), older, female, and BMI ≥ 24.0 kg/m^2^ were significantly associated with an increased risk for mental health difficulties. The probability of having mental health difficulties for those whose maternal education was senior high school dropped by 83.7% (OR, 0.163, [95%CI, 0.032-0.825]) and university and above dropped by 76.8% (OR, 0.232, [95% CI, 0.058-0.928]), compared with maternal education with junior high school and below (Figure 1C).

Factors affecting mental health also vary between different sexes. For boys, extended sleep duration and paternal education with junior high school and below were risk factors for mental health difficulties (Figure 1D). For girls, the older age was a protective factor (OR, 0.854, [95% CI, 0.756-0.965]). The risks of mental health difficulties with 1-2 h/d and ≥2 h/d screen time on weekends were up to 1.899 times and 3.420 times, compared to those with less than 1h/d, respectively (OR, 1.899, [95%CI, 1.066-3.386]; OR, 3.420, [95%CI, 1.923-6.081]) (Figure 1E).

### Associations between five dimensions and effect factors

**Figure 2:**
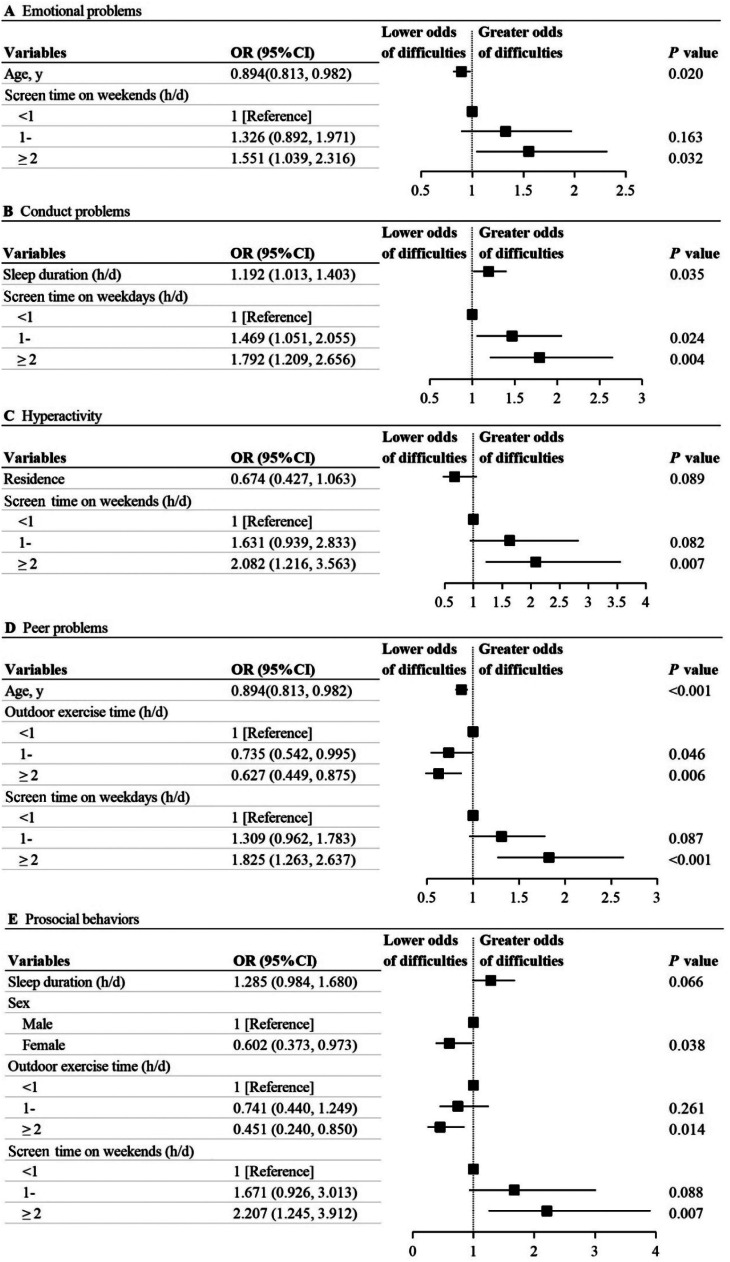
Associations between five dimensions and influencing factors.

Age and ≥ 2h/d screen time on weekends were significantly associated with emotional problems (OR, 0.894, [95%CI, 0.813-0.982]; OR, 1.551, [95%CI, 1.039-2.316]). Sleep duration and screen time on weekdays were significantly associated with an increased risk for conduct problems (OR, 1.192, [95%CI, 1.013-1.403]; “1-” h/d vs “< 1” h/d: OR, 1.469, [95% CI, 1.051-2.055]; “≥ 2” h/d vs “< 1” h/d: OR, 1.792, [95%CI, 1.209-2.656]). Screen time on weekends of ≥ 2h/d was associated with greater odds of hyperactivity, compared with screen time of < 1 h/d (OR, 2.082, [95%CI, 1.216-3.563]). Age (OR, 0.894, [95%CI, 0.813-0.982]), outdoor exercise time (“1-” h/d vs “< 1” h/d: OR, 0.735, [95% CI, 0.542-0.995]; “≥ 2” h/d vs “< 1” h/d: OR, 0.627, [95%CI, 0.449-0.875]) were associated with lower odds of peer problems. Screen time on weekdays of ≥ 2 h/d was associated with higher odds of peer problems, compared with <1 h/d screen time on weekdays (OR, 1.825, [95%CI, 1.263-2.637]). The factors associated with prosocial behaviors were sex (female vs male: OR, 0.602, [95%CI, 0.373-0.973]), ≥ 2 h/d outdoor exercise time (“≥ 2” h/d vs “< 1” h/d: OR, 0.451, [95%CI, 0.240-0.850]) and ≥ 2 h/d screen time on weekends (“≥ 2” h/d vs “< 1” h/d: OR, 2.207, [95%CI, 1.245-3.912]) (Figure 2)

### Associations between SDQ scores and effect factors

According to the results of the multiple linear regression analysis, < 1 h/d outdoor exercise time (“1-” h/d vs “< 1” h/d: *β*, -0.720, [95% CI, -1.422– -0.018.]; “≥ 2” h/d vs “< 1” h/d: *β*, -0.761, [95%CI, -1.510– -0.012]), ≥ 2 h/d screen time on weekdays (“≥ 2” h/d vs “< 1” h/d: *β*, 1.376, [95%CI, 0.437-2.315]) and ≥ 1 h/d screen time on weekends (“1-” h/d vs “< 1” h/d: *β*, 0.861, [95% CI, 0.160-1.562]; “≥ 2” h/d vs “< 1” h/d: *β*, 1.341, [95%CI, 0.558-2.124]) were associated with higher scores of SDQ, respectively. Factors associated with emotional problems, conduct, hyperactivity, peer problems, and prosocial behaviors are shown in Table 2. Likewise, multiple linear regression was used to explore factors associated with scores of SDQ in different age groups and sexes, respectively (Supplementary Tables 1- 4).

**Table 2:** Associations of related factors with scores of SDQ [*β* (95%CI)].

Variables		Total difficulties score	Emotional problems	Conduct	Hyperactivity	Peer problems	Prosocial behaviors
Sleep duration, h/d		NA	-0.151 (-0.289, -0.013)*	NA	NA	NA	NA
Sex	Male	NA	1 [Reference]	NA	NA	NA	1 [Reference]
	Female	NA	0.361 (0.123, 0.600)*	NA	NA	NA	0.301 (0.080, 0.522)*
Only child	Yes	NA	NA	NA	NA	NA	1 [Reference]
	No	NA	NA	NA	NA	NA	0.263 (-0.006, 0.531)
Residence	Rural	NA	NA	NA	NA	NA	1 [Reference]
	Urban	NA	NA	NA	NA	NA	0.221 (-0.012, 0.454)
Paternal education	Junior high school and below	NA	NA	NA	1 [Reference]	NA	NA
	Senior high school	NA	NA	NA	-0.388 (-0.649, -0.127)*	NA	NA
	University and above	NA	NA	NA	-0.349 (-0.655, -0.043)*	NA	NA
Maternal education	Junior high school and below	NA	1 [Reference]	1 [Reference]	NA	NA	NA
	Senior high school	NA	-0.232 (-0.504, 0.039)	0.238 (0.041, 0.435)*	NA	NA	NA
	University and above	NA	NA	NA	NA	NA	NA
Outdoor exercise time, h/d	< 1	1 [Reference]	1 [Reference]	NA	NA	NA	1 [Reference]
	1-	-0.720 (-1.422, -0.018)*	-0.248 (-0.536, 0.039)	NA	NA	NA	NA
	≥ 2	-0.761 (-1.510, -0.012)*	-0.285 (-0.593, 0.022)	NA	NA	NA	0.398 (0.156, 0.640)*
Screen time on weekdays, h/d	< 1	1 [Reference]	NA	1 [Reference]	1 [Reference]	1 [Reference]	1 [Reference]
	1-	NA	NA	0.300 (0.093, 0.507)*	NA	0.176 (-0.008, 0.360)	-0.454 (-0.729, -0.179)*
	≥ 2	1.376 (0.437, 2.315)*	NA	0.595 (0.341, 0.850)*	0.455 (0.088, 0.822)*	0.402 (0.176, 0.629)*	-0.444 (-0.819, -0.070)*
Screen time on weekends, h/d	< 1	1 [Reference]	1 [Reference]	NA	1 [Reference]	NA	1 [Reference]
	1-	0.861 (0.160, 1.562)*	0.266(-0.019,0.551)	NA	0.381 (0.106, 0.655)*	NA	NA
	≥ 2	1.341 (0.558, 2.124)*	0.456(0.162,0.751)*	NA	0.713 (0.405, 1.021)*	NA	-0.306 (-0.587, -0.024)*

NA, not applicable.

⁎ *p <* 0.05.

## Discussion

The present cross-sectional study examined mental health difficulties and related factors in Chinese children and adolescents during the COVID-19 pandemic. The overall prevalence of mental health difficulties was 12.98%. The study indicated that younger, females and ≥2 h/d screen time on weekdays were significantly associated with greater odds of mental health difficulties in the entire study population during the COVID-19 pandemic. For children, outdoor exercise time and screen time on weekdays were found to be influencing factors for mental health difficulties. The factors associated with mental health difficulties were age, sex, BMI, and maternal education in adolescents. On the other hand, sleep duration and paternal education were associated with mental health difficulties in males, but age and screen time on weekends were related factors in females.

The prevalence of mental health difficulties among children and adolescents during the COVID-19 pandemic was higher than the reported 9.8% in a 2017 study during the pre-pandemic period.^18^ The present research showed that a relatively high proportion of children and adolescents suffered from psychological problems during the pandemic and it is likely that the pandemic is worsening the mental health of youth. Stress is usually generated by external or internal and causes physiological or psychological disorders in the body.^19^ Existing evidence supports that the COVID-19 pandemic acts as a stressor and triggers a series of mental health problems among children and adolescents.^20^ In addition, the central nervous system is in a vulnerable developmental window in children or even adolescents. Any stressful challenges may have a short or even long-term negative psychological impact on children and adolescents.^21^ These factors may be related to the increase in psychological problems during the pandemic. A large-scale survey, which involved a million scale children and adolescents in Guangzhou, reported that the prevalence of psychological distress was 10.5% during the pandemic.^13^ Explanations for the difference may be due to sample size, methods or scales used.

The present study found that age, sex and screen time on weekdays were significantly associated with mental health difficulties. The majority of studies suggested that girls seem to be more affected by the psychological effects during the pandemic.^22^^,^^23^ Consistent with current evidence, the authors observed that girls scored higher than boys in emotional problems. This may be due to the fact that girls are more emotional, delicate and sensitive than boys. Girls are more vulnerable to mental health problems when they face unexpected challenges. Tso et al. showed that excessive time on electronic devices was found to be a risk factor in children aged 6-12 years.^24^ Xiang et al. reported that children and adolescents with leisure screen time of more than 2 hours increased by 24% during the COVID-19 pandemic.^25^ Although the association between outdoor exercise time and mental health difficulties was not observed, the trend of sufficient outdoor exercise time with lower risks of peer problems and abnormal prosocial behaviors existed among participants. Positive interactions with peers were increased through outdoor exercise, which could promote positive mental health among children and adolescents.^26^ Therefore, mental health problems control efforts should focus on the appropriate use of electronic devices and prolonged outdoor exercise time.

Notably, the factors associated with mental health difficulties varied by different age groups and sexes during the COVID-19 pandemic. For children, this study indicates screen time on weekdays and outdoor exercise time as factors contributing to differences in mental health difficulties. While older, female, overweight/obese, and maternal education with junior high school and below were found to be risk factors in adolescents. This suggests that children are more vulnerable to excessive screen time and lack of outdoor exercise time, leading to a high risk of mental health difficulties. Younger children are yet physically and mentally mature and are very vulnerable to external influences. Prolonged exposure to the virtual world created by electronic products can easily lead to a disconnection from the real world, which is detrimental to their psychological development. In addition, overweight/obese adolescents with poor body image may experience greater exposure to stressors, which aggravate the harm to mental health.^27^ A previous study showed that a higher educational level of mothers is associated with fewer psychological issues in adolescents, which is consistent with these results.^10^ This may be related to the prevailing family pattern in China, where mothers spend much more time with their children than fathers and interact with their children more than fathers do. Future studies need larger sample sizes to explore why maternal education is relevant primarily in adolescents and not in children. This study also found that boys were more vulnerable to sleep duration and paternal education, while the most important determinants of mental health difficulties were age and screen time on weekends for girls.

This study is different from previous studies which only explored factors associated with mental health status based on an entire population of children and adolescents. Subgroup analyses were conducted to assess differences in factors influencing mental health status between different age groups and sexes. Additionally, the SDQ, a screening questionnaire targeted children and adolescents, assessed mental health status on five dimensions. However, several limitations need to be addressed. First, the cross-sectional study resulting in causation cannot be addressed. Second, although numerous relevant factors have been included in this study, residual confounding may still exist. Third, excluding eligible children (children with missing data) and the sample based on convenience and not equal probability may inevitably introduce selection bias. Finally, the study population was from northern China, which limited the generalizability of the findings to other populations.

## Conclusion

The prevalence of mental health difficulties among children and adolescents was relatively high during the COVID-19 pandemic, with mental health difficulties being more likely to be younger, female and excessive screen time on weekdays, especially the factors associated with mental health difficulties varied by different age groups and sexes. Therefore, it is crucial for government, healthcare planners, teachers and parents to pay attention to the mental health among children and adolescents during and beyond the COVID-19 pandemic and take specific measures for different age groups and sexes to mitigate the impact.

## Conflicts of interest

The authors declare no conflicts of interest.

## Appendix Supplementary materials

Image, application 1

## Footnotes

Supplementary material associated with this article can be found in the online version at doi:10.1016/j.jped.2024.03.004.
