# Supplementary material for: Mental health difficulties and related factors in Chinese children and adolescents during the COVID-19 pandemic: a cross-sectional study
Source: J Pediatr (Rio J). 2024 Apr 18;100(4):413–21. doi: 10.1016/j.jped.2024.03.004 (PMC11331231; doi:10.1016/j.jped.2024.03.004)
Supplement: Supplementary file 1 [file mmc1.docx]

**JPED-D-23-00492 – Supplemental Online Content**

**
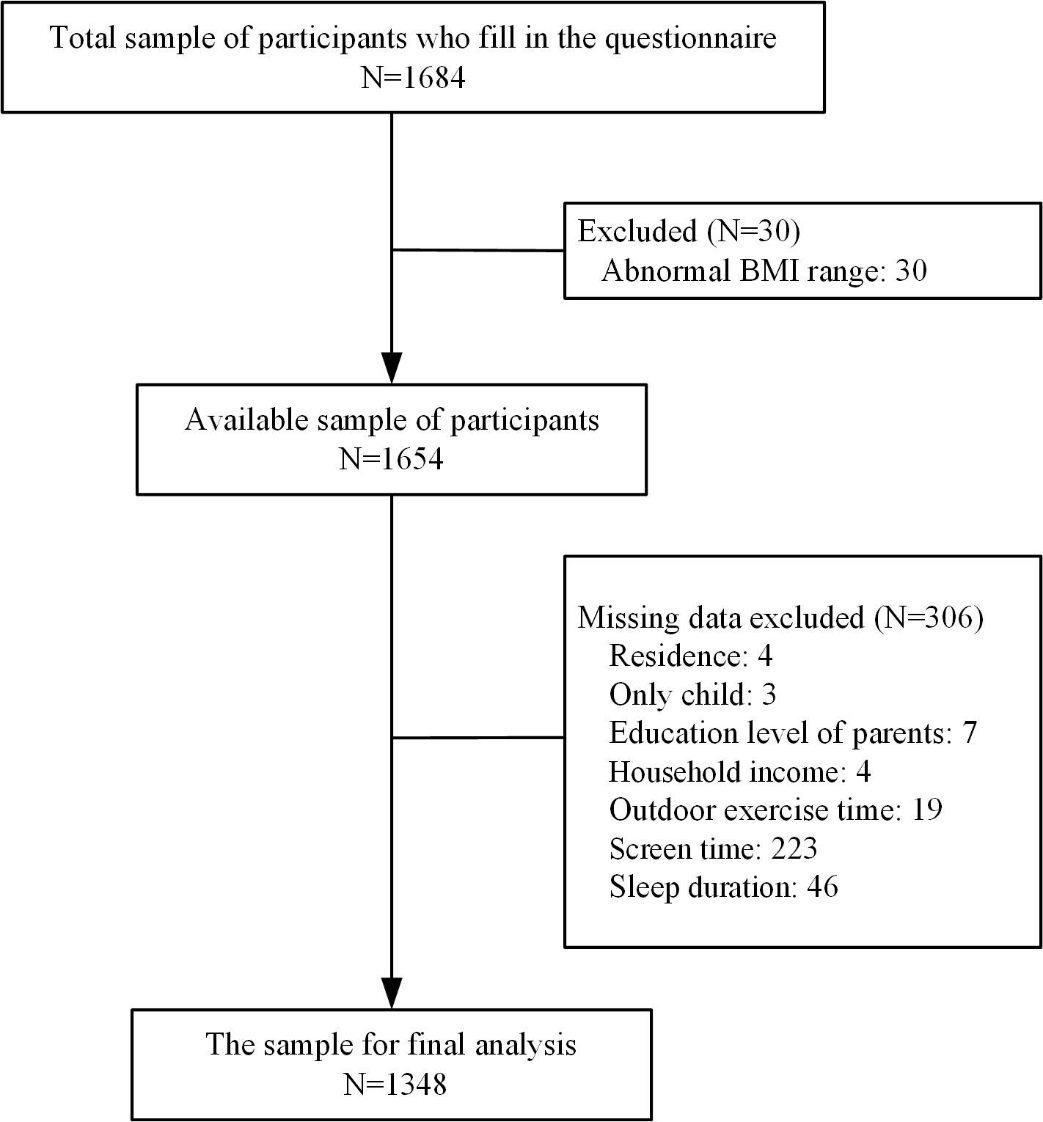
**

**Supplementary Figure 1** Flow diagram of the enrolment in this study.

**Supplementary Table 1** Associations of associated factors with scores of SDQ in children [*β* (95%CI)].

| **Variables** | | **Total difficulties score** | **Emotional problems** | **Conduct** | **Hyperactivity** | **Peer problems** | **prosocial behaviors** |
| --- | --- | --- | --- | --- | --- | --- | --- |
| Age, y |  | NA | -0.094 (-0.192, 0.005) | NA | NA | NA | -0.079 (-0.171, 0.013) |
| Sleep duration, h/d |  | NA | NA | NA | -0.148 (-0.310, 0.014) | NA | NA |
| Sex | Male | NA | 1 [Reference] | NA | NA | NA | 1 [Reference] |
|  | Female | NA | 0.322 (0.069, 0.575)^*^ | NA | NA | NA | 0.311 (0.076, 0.546)^*^ |
| Paternal education | Junior high school and below | 1 [Reference] | NA | NA | 1 [Reference] | NA | NA |
|  | Senior high school | -0.714 (-1.382, -0.046)^*^ | NA | NA | -0.406 (-0.671, -0.142)^*^ | NA | NA |
|  | University and above | NA | NA | NA | NA | NA | NA |
| Maternal education | Junior high school and below | NA | 1 [Reference] | 1 [Reference] | NA | NA | NA |
|  | Senior high school | NA | -0.268 (-0.558, 0.022) | 0.273 (0.061,0.486)^*^ | NA | NA | NA |
|  | University and above | NA | NA | NA | NA | NA | NA |
| Outdoor exercise time, h/d | <1 | 1 [Reference] | NA | NA | NA | 1 [Reference] | 1 [Reference] |
|  | 1- | -0.783 (-1.534, -0.031)^*^ | NA | NA | NA | -0.198 (-0.391, -0.004)^*^ | NA |
|  | ≥2 | -0.936 (-1.737, -0.134)^*^ | NA | NA | NA | -0.191 (-0.397, 0.016) | 0.469 (0.212, 0.725)^*^ |
| Screen time on weekdays, h/d | <1 | 1 [Reference] | NA | 1 [Reference] | 1 [Reference] | 1 [Reference] | 1 [Reference] |
|  | 1- | 1.124 (0.383, 1.865)^*^ | NA | 0.316 (0.096, 0.535)^*^ | NA | 0.209 (0.018, 0.400)^*^ | -0.444 (-0.733, -0.155)^*^ |
|  | ≥2 | 2.400 (1.406, 3.394)^*^ | NA | 0.671 (0.377, 0.965)^*^ | 0.500 (0.076, 0.925)^*^ | 0.350 (0.094, 0.606)^*^ | -0.401 (-0.822, 0.021) |
| Screen time on weekends, h/d | <1 | NA | 1 [Reference] | NA | 1 [Reference] | NA | 1 [Reference] |
|  | 1- | NA | 0.255 (-0.037, 0.547) | NA | 0.368 (0.080, 0.657)^*^ | NA | NA |
|  | ≥2 | NA | 0.456 (0.140, 0.773)^*^ | NA | 0.654 (0.309, 0.999)^*^ | NA | -0.355 (-0.666, -0.045)^*^ |

NA, not applicable.

^*^: p < 0.05.

**Supplementary Table 2** Associations of associated factors with scores of SDQ in adolescents [*β* (95%CI)].

| **Variables** | | **Total difficulties score** | **Emotional problems** | **Conduct** | **Hyperactivity** | **Peer problems** | **prosocial behaviors** |
| --- | --- | --- | --- | --- | --- | --- | --- |
| Age, y |  | NA | NA | 0.186 (0.019, 0.354)^*^ | NA | NA | NA |
| BMI, No. (%), kg/m^2^ | <24.0 | NA | NA | NA | NA | 1 [Reference] | NA |
|  | ≥24.0 | NA | NA | NA | NA | 0.769 (-0.115, 1.652) | NA |
| Sex | Male | NA | 1 [Reference] | NA | NA | NA | NA |
|  | Female | NA | 0.734 (-0.003, 1.471) | NA | NA | NA | NA |
| Only child | Yes | NA | NA | NA | NA | NA | 1 [Reference] |
|  | No | NA | NA | NA | NA | NA | 0.643 (-0.028, 1.313) |
| Residence | Rural | NA | 1 [Reference] | NA | NA | NA | NA |
|  | Urban | NA | 0.916 (0.058, 1.773)^*^ | NA | NA | NA | NA |
| Paternal education | Junior high school and below | 1 [Reference] | NA | NA | 1 [Reference] | NA | NA |
|  | Senior high school | 1.946 (-0.008, 3.899) | NA | NA | NA | NA | NA |
|  | University and above | NA | NA | NA | -0.741 (-1.388, -0.144)^*^ | NA | NA |
| Maternal education | Junior high school and below | NA | NA | NA | NA | NA | NA |
|  | Senior high school | NA | NA | NA | NA | NA | NA |
|  | University and above | NA | NA | NA | NA | NA | NA |
| Outdoor exercise time, h/d | <1 | NA | NA | 1 [Reference] | NA | NA | NA |
|  | 1- | NA | NA | -0.465 (-0.988, 0.057) | NA | NA | NA |
|  | ≥2 | NA | NA | NA | NA | NA | NA |
| Screen time on weekdays, h/d | <1 | NA | NA | NA | NA | 1 [Reference] | NA |
|  | 1- | NA | NA | NA | NA | NA | NA |
|  | ≥2 | NA | NA | NA | NA | 0.548 (-0.002, 1.098) | NA |
| Screen time on weekends, h/d | <1 | 1 [Reference] | NA | NA | 1 [Reference] | NA | NA |
|  | 1- | NA | NA | NA | NA | NA | NA |
|  | ≥2 | 1.724 (-0.043, 3.491) | NA | NA | 1.075 (0.455, 1.696)^*^ | NA | NA |

NA, not applicable; BMI, body mass index.

^*^: p < 0.05.

**Supplementary Table 3** Associations of associated factors with scores of SDQ in male [*β* (95%CI)].

| **Variables** | | **Total difficulties score** | **Emotional problems** | **Conduct** | **Hyperactivity** | **Peer problems** | **prosocial behaviors** |
| --- | --- | --- | --- | --- | --- | --- | --- |
| Age, y |  | NA | NA | NA | NA | NA | -0.083 (-0.175, 0.009) |
| Sleep duration, h/d |  | NA | NA | 0.119 (-0.020, 0.258) | NA | NA | NA |
| Residence | Rural | NA | NA | NA | NA | 1 [Reference] | 1 [Reference] |
|  | Urban | NA | NA | NA | NA | -0.200 (-0.411, 0.010) | 0.399 (0.092, 0.706)^*^ |
| Paternal education | Junior high school and below | NA | 1 [Reference] | NA | 1 [Reference] | NA | NA |
|  | Senior high school | NA | -0.404 (-0.752, -0.057)^*^ | NA | -0.456 (-0.825, -0.087)^*^ | NA | NA |
|  | University and above | NA | NA | NA | -0.658 (-1.077, -0.238)^*^ | NA | NA |
| Maternal education | Junior high school and below | NA | NA | 1 [Reference] | NA | 1 [Reference] | NA |
|  | Senior high school | NA | NA | 0.370 (0.095, 0.645)^*^ | NA | -0.214 (-0.456, 0.028) | NA |
|  | University and above | NA | NA | NA | NA | NA | NA |
| Outdoor exercise time, h/d | <1 | NA | 1 [Reference] | NA | NA | 1 [Reference] | 1 [Reference] |
|  | 1- | NA | -0.371 (-0.753, 0.012) | NA | NA | -0.286 (-0.538, -0.034)^*^ | NA |
|  | ≥2 | NA | -0.431 (-0.846, -0.017)^*^ | NA | NA | -0.309 (-0.582, -0.037)^*^ | 0.564 (0.234, 0.895)^*^ |
| Screen time on weekdays, h/d | <1 | 1 [Reference] | NA | 1 [Reference] | 1 [Reference] | 1 [Reference] | 1 [Reference] |
|  | 1- | NA | NA | NA | NA | NA | -0.481 (-0.857, -0.104)^*^ |
|  | ≥(2 | 1.893 (0.741, 3.045)^*^ | NA | 0.461 (0.115, 0.807)^*^ | 0.554 (0.050, 1.058)^*^ | 0.431 (0.126, 0.735)^*^ | -0.658 (-1.160, -0.157)^*^ |
| Screen time on weekends, h/d | <1 | NA | NA | NA | 1 [Reference] | NA | 1 [Reference] |
|  | 1- | NA | NA | NA | NA | NA | NA |
|  | ≥2 | NA | NA | NA | 0.459 (0.083, 0.834)^*^ | NA | -0.405 (-0.773, -0.037)^*^ |

NA, not applicable.

^*^ *p* < 0.05.

**Supplementary Table 4** Associations of associated factors with scores of SDQ in female [*β* (95%CI)].

| **Variables** | | **Total difficulties score** | **Emotional problems** | **Conduct** | **Hyperactivity** | **Peer problems** | **prosocial behaviors** |
| --- | --- | --- | --- | --- | --- | --- | --- |
| Age, y |  | NA | NA | -0.073 (-0.142, -0.003)^*^ | -0.116 (-0.207, -0.025)^*^ | NA | NA |
| Sleep duration, h/d |  | NA | -0.283 (-0.484, -0.082)^*^ | NA | NA | NA | NA |
| Residence | Rural | NA | 1 [Reference] | NA | NA | 1 [Reference] | NA |
|  | Urban | NA | 0.411 (0.019, 0.802)^*^ | NA | NA | 0.234 (-0.001, 0.468) | NA |
| Paternal education | Junior high school and below | NA | NA | NA | NA | NA | 1 [Reference] |
|  | Senior high school | NA | NA | NA | NA | NA | NA |
|  | University and above | NA | NA | NA | NA | NA | -0.437 (-0.850, -0.024)^*^ |
| Maternal education | Junior high school and below | NA | 1 [Reference] | NA | NA | NA | 1 [Reference] |
|  | Senior high school | NA | -0.480 (-0.918, -0.042)^*^ | NA | NA | NA | -0.368 (-0.730, -0.005)^*^ |
|  | University and above | NA | -0.646 (-1.149, -0.143)^*^ | NA | NA | NA | NA |
| Household income, | <3000 | NA | NA | NA | NA | 1 [Reference] | NA |
| CNY | 3000-10000 | NA | NA | NA | NA | NA | NA |
|  | >10000 | NA | NA | NA | NA | -0.320 (-0.604, -0.036)^*^ | NA |
| Outdoor exercise time, h/d | <1 | NA | NA | NA | 1 [Reference] | NA | 1 [Reference] |
|  | 1- | NA | NA | NA | -0.332 (-0.674, 0.010) | NA | 0.392 (0.006, 0.778)^*^ |
|  | ≥2 | NA | NA | NA | NA | NA | 0.452 (0.048, 0.856)^*^ |
| Screen time on weekdays, h/d | <1 | 1 [Reference] | NA | 1 [Reference] | NA | 1 [Reference] | 1 [Reference] |
|  | 1- | 1.026 (-0.131, 2.182)^*^ | NA | 0.436 (0.136, 0.737)^*^ | NA | 0.259 (-0.011, 0.529) | -0.450 (-0.822, -0.078)^*^ |
|  | ≥2 | 1.621 (0.065, 3.177)^*^ | NA | 0.798 (0.420, 1.176)^*^ | NA | 0.352 (0.017, 0.688)^*^ | NA |
| Screen time on weekends, h/d | <1 | 1 [Reference] | 1 [Reference] | NA | 1 [Reference] | NA | NA |
|  | 1- | 1.183 (0.075, 2.291)^*^ | 0.443 (0.017, 0.869)^*^ | NA | 0.729 (0.339, 1.119)^*^ | NA | NA |
|  | ≥2 | 1.786 (0.436, 3.137)^*^ | 0.785 (0.334, 1.235)^*^ | NA | 1.244 (0.828, 1.661)^*^ | NA | NA |

NA, not applicable; CNY, Chinese Yuan.

^*^: p < 0.05.
